# Radiation-Induced Endothelial Ferroptosis Accelerates Atherosclerosis via the DDHD2-Mediated Nrf2/GPX4 Pathway

**DOI:** 10.3390/biom14070879

**Published:** 2024-07-22

**Authors:** Xi Su, Feng Liang, Ya Zeng, Zhang-Ru Yang, Yue-Zhen Deng, Yun-Hua Xu, Xu-Wei Cai

**Affiliations:** 1 Department of Radiation Oncology, Shanghai Chest Hospital, Shanghai Jiao Tong University School of Medicine, Shanghai 200030, China; suxi97@alumni.sjtu.edu.cn (X.S.); yzr2296@shchest.org (Z.-R.Y.); 2 Department of Cardiology, Shanghai Chest Hospital, Shanghai Jiao Tong University School of Medicine, Shanghai 200030, China; liangfengwind@sjtu.edu.cn; 3 Shanghai Institute of Thoracic Oncology, Shanghai Chest Hospital, Shanghai Jiao Tong University School of Medicine, Shanghai 200030, China; 4 Shanghai Lung Cancer Center, Shanghai Chest Hospital, Shanghai Jiao Tong University School of Medicine, Shanghai 200030, China

**Keywords:** radiation-associated atherosclerosis, ferroptosis, endothelial injury, DDHD2, Nrf2/GPX4

## Abstract

This study sought to explore potential roles of endothelial ferroptosis in radiation-associated atherosclerosis (RAA) and molecular mechanisms behind this phenomenon. Here, an in vivo RAA mouse model was used and treated with ferroptosis inhibitors. We found that the RAA group had a higher plaque burden and a reduction in endothelial cells with increased lipid peroxidation compared to the control group, while ameliorated by liproxstatin-1. In vitro experiments further confirmed that radiation induced the occurrence of ferroptosis in human artery endothelial cells (HAECs). Then, proteomics analysis of HAECs identified domain-containing protein 2 (DDHD2) as a co-differentially expressed protein, which was enriched in the lipid metabolism pathway. In addition, the level of lipid peroxidation was elevated in DDHD2-knockdown HAECs. Mechanistically, a significant decrease in the protein and mRNA expression of glutathione peroxidase 4 (GPX4) was observed in HAECs following DDHD2 knockdown. Co-immunoprecipitation assays indicated a potential interaction between DDHD2 and nuclear factor erythroid 2-related factor 2 (Nrf2). The downregulation of Nrf2 protein was also detected in DDHD2-knockdown HAECs. In conclusion, our findings suggest that radiation-induced endothelial ferroptosis accelerates atherosclerosis, and DDHD2 is a potential regulatory protein in radiation-induced endothelial ferroptosis through the Nrf2/GPX4 pathway.

## 1. Introduction

Radiotherapy, an important approach for treating tumor diseases, can also cause damage to blood vessels within the radiation field, increasing the risk of cardiovascular disease [1,2]. Previous studies have reported a notable rise in plaques incidence and intima-media thickness in irradiated carotid arteries among patients with head and neck cancer [3,4]. Radiation-associated atherosclerosis (RAA) is a serious complication of radiotherapy in cancer patients owing to death from stroke and risk of cardiac ischemia [5,6]. It is characterized by an inflammatory plaque phenotype prone to hemorrhage [7,8,9]. Although research on the mechanism of RAA is still limited, it is well-established that sustained inflammation induced by irradiation significantly contributes to the onset and progression of RAA [8,9,10]. Endothelial cells are susceptible and sensitive to irradiation. Endothelial injury is regarded as a trigger of inflammation in radiation-induced organ damage [11]. Radiation exposure activates inflammatory signaling pathways in endothelial cells, leading to increased expression of adhesion molecules that attract immune cells, exacerbating inflammation [12,13]. However, the mechanism of irradiation-induced endothelial injury and subsequent inflammation in RAA remains unclear. 

Ferroptosis is an iron-dependent form of regulated cell death induced by excessive lipid peroxidation, which differs from apoptosis and necrosis [14]. Iron plays a pivotal role in ferroptosis by triggering the Fenton reaction and activating iron-containing enzymes, causing reactive oxygen species (ROS) production. Then, ROS reacts with phospholipids containing polyunsaturated fatty acids (PUFAs), leading to lipid peroxidation. The accumulation of lipid peroxidation and its metabolite, such as 4-hydroxynonenal (4-HNE), can cause membrane instability and permeabilization, ultimately resulting in cell death [15,16]. Previous studies revealed that radiation exposure can trigger ferroptosis, which plays an important role in radiation-mediated injuries, such as radiation-induced lung injury [17] and intestinal injuries [18]. Notably, emerging evidence has linked ferroptosis to cardiovascular diseases, including atherosclerosis and cardiomyopathy [19,20,21]. Bai et al. [22] also revealed that endothelial ferroptosis induced by oxidized low-density lipoprotein (ox-LDL) could accelerate atherosclerosis in *ApoE*−/− mice. Thus, we hypothesize that radiation-induced endothelial ferroptosis may be a potential mechanism contributing to the acceleration of RAA. 

In this study, the RAA mouse model and irradiated HAECs were established and treated with ferroptosis inhibitors. Then, four-dimension label-free quantification (4D-LFQ) proteomics was used to identify key proteins associated with radiation-induced endothelial ferroptosis. Moreover, we preliminary explored the molecular mechanism of the key protein identified in this process. Hopefully, our study can contribute to a deeper understanding of the mechanisms underlying RAA and provide a novel therapeutic target for RAA.

## 2. Materials and Methods

### 2.1. Animals and Irradiation Protocols

**Figure 1:**
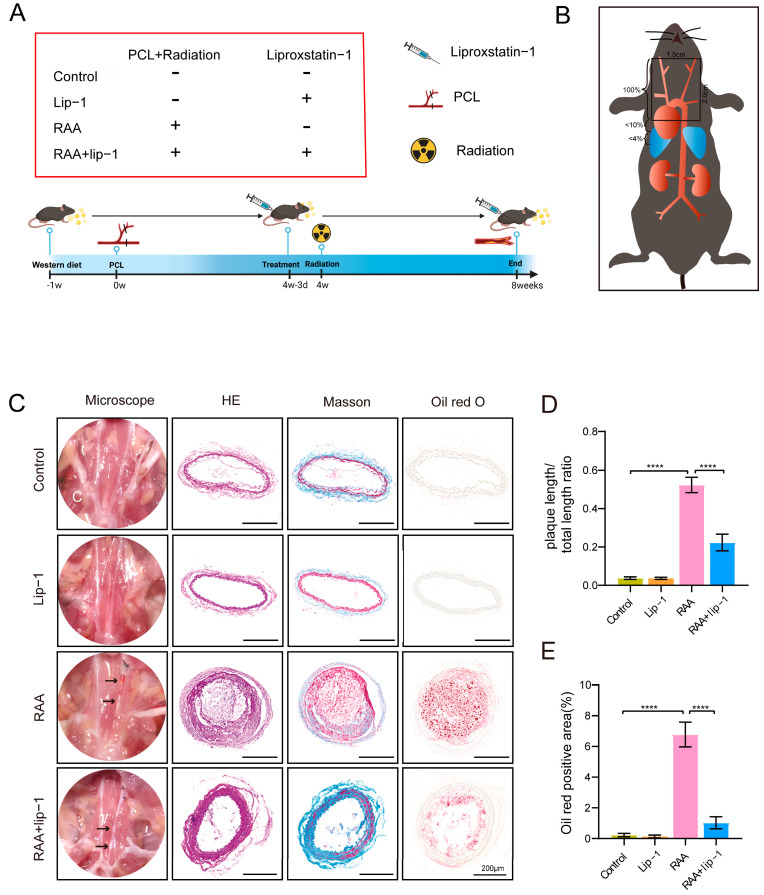
Ferroptosis inhibitor alleviates radiation-induced atherosclerosis lesions. (**A**) The study design scheme illustrates groups with treatment. (**B**) The irradiation field in terms of anatomic overview (2 × 1.5 cm^2^). (**C**) Representative images of microscopy and sections stained with hematoxylin and eosin, Masson, and Oil Red of the left carotid artery. (**D**) Quantification of the length of carotid atherosclerotic plaque from *ApoE*−/− mice in each group (*n* = 8). (**E**) Quantification of the oil red positive area of carotid atherosclerotic plaque from *ApoE*−/− mice in each group (*n* = 8). **** *p* < 0.0001.

Male *ApoE*−/− mice (C57BL/6J background, 6–8 weeks) were purchased from GemPharmatech Co Ltd. A Western diet containing 50% carbohydrates, 21% fat, and 20% protein was given to all mice. The RAA mouse model was established by irradiating *ApoE*−/− mice with partial ligation of the left common carotid artery (LCCA), as previously described [9,23,24]. Briefly, *ApoE*−/− mice were randomly assigned to 4 groups: control group, liproxstatin-1 (Lip-1) group, RAA group, and RAA+lip-1 group. The control group received a sham operation and sham radiation. The RAA group and RAA+lip-1 group received a partial ligation of LCCA and radiation in the 4th week, and the Lip-1 group and the RAA+lip-1 group received a ferroptosis inhibitor Lip-1 (intraperitoneally administered, 10 mg/kg/d) treatment three times before radiation followed by daily injection until 4 weeks post-radiation (Figure 1A). Mice in the other three groups received an equal volume of normal saline at the same time, as previously described. The Lip-1 (HY-12726, MCE) was dissolved in dimethyl sulfoxide (DMSO) and diluted in normal saline according to the manufacturer’s protocol. 

The irradiation procedure was performed according to a previous study [23]. Mice were treated with image-guided radiotherapy via the Small Animal Radiation Research Platform (SARRP, Xstrahl, Walsall, UK). Briefly, mice were irradiated with a single dose of 10 Gy or 0 Gy (sham radiation) to the neck region at a dose rate of 2.4 Gy/min, with a voltage of 220 kV, a current of 13 mA, and copper filtering. The field area was 20 × 15 mm and included carotid arteries and the aortic arch, as previously described [23] (Figure 1B). Mice were sacrificed 4 weeks after irradiation and the LCCA and serum were collected.

### 2.2. Cell Culture and Treatment

The human artery endothelial cell line HAECs (ATCC, PCS-100-011) and 293T (Shanghai Institute of Cell Biology of Chinese Academy of Sciences, Shanghai, SCSP-502) cells were cultured in a 37 °C incubator with 5% CO_2_. The ferrostatin-1 (Fer-1) (Sigma, Cream Ridge, NJ, USA, SML0583) and the iron chelator deferoxamine (DFO) (Sigma, D9533) were purchased from Sigma. DFO is an iron chelator that prevents ferroptosis by sequestering iron [15,25]. To determine the effects of ferroptosis inhibitors on irradiated HAECs, the HAECs were pretreated with 2–5 μM Fer-1 or 10–20 μM DFO or an equal volume of DMSO for 24 h as well as two days after radiotherapy. A single dose (0, 4, 8, 16 Gy) in vitro irradiation was delivered to HAECs (SARRP, 2.4 Gy/min). Both Fer-1 and Lip-1 are reported to be ferroptosis inhibitors that can prevent ferroptosis by trapping chain-carrying radicals and inhibiting lipid peroxidation, while Lip-1 was more stable in vivo than Fer-1 [26]. 

### 2.3. RNA Extraction and Real-Time Quantitative PCR

Total RNA was extracted from HAECs with an RNA-easy Isolation Reagent (Vazyme, Nanjing, China, R701-02). An amount of 1 μg of RNA was reverse-transcribed to cDNA. The cDNA was used as a template for real-time quantitative PCR with the AceQ^®^ Universal SYBR^®^ qPCR Master Mix (Vazyme, Nanjing, Q511-02) according to the manufacturer’s protocol. β-actin was used as an internal control and the 2^−ΔΔCt^ method was utilized to analyze the relative change. All primers were designed and synthesized by Sangon Biotech (Shanghai, China). The primers used are listed in the supplementary material (Supplementary Table S1). 

### 2.4. Western Blot

Cellular proteins were extracted, underwent electrophoretic separation, and were subsequently transferred to a polyvinylidene fluoride membrane. Then, the membranes were separately incubated overnight at 4 °C with the following primary antibodies: glyceraldehyde-3-phosphate dehydrogenase (GAPDH, CST, USA, 2118, 1:2000), domain-containing protein 2 (DDHD2, Proteintech, Wuhan, China, 25203-1-AP), glutathione peroxidase 4 (GPX4, Abmart, T56959, 1:2000), anti-his antibody (Proteintech, Wuhan, 66005-1-Ig), anti-flag antibody (proteintech, Wuhan, 66008-4-Ig). After being washed three times, the membranes were incubated with secondary antibodies for 1 h at room temperature. Proteins were visualized with chemiluminescence using LumiBlueTM ECL Extra (Sharebio, Shanghai, China, AB270229).

### 2.5. Tissue Preparation and Histological Studies

Carotid arteries were fixed in 4% paraformaldehyde for 4 h and subsequently immersed in 30% sucrose overnight at 4 °C. Then, tissues were embedded and frozen using an optimal cutting temperature compound (Sakura Finetek, Tokyo, Japan). Fixed carotid arteries were sectioned (8 μm) and stained with Masson’s trichrome staining, hematoxylin and eosin (H&E) staining, and oil red staining (Servicebio, Wuhan, China). Section images were captured under light microscopy (Nikon, Tokyo, Japan).

### 2.6. Immunofluorescence

The frozen sections were incubated with donkey serum for 1 h and then incubated with primary antibodies against platelet endothelial cell adhesion molecule-1 (CD31, AF3628, 5 μg/mL, R&D System, Minneapolis, MN, USA), 4-HNE (ab46545, 1:200, Abcam, Cambridge, UK), interleukin-1β (IL-1β, ab234437, 1:200, Abcam, UK) and tumor necrosis factor-α (TNF-α, ab300093, 1:200, Abcam, UK) at 4 °C overnight. After the sections were washed thrice, they were incubated with secondary antibodies for 1 h. The nuclei were counterstained with DAPI (G1012-10ML, Servicebio, Wuhan, China).

### 2.7. Transmission Electron Microscopy (TEM)

HAECs were washed with PBS and fixed with 2.5% electron microscope-grade glutaraldehyde fixing solution (G1102, Servicebio, Wuhan, China) for 30 min at room temperature, and then incubated overnight at 4 °C. Following fixation with 0.1% osmium tetroxide, the samples were dehydrated through a gradient of escalating ethanol and propylene oxide concentrations. Subsequently, the specimens were embedded, sectioned into ultrathin slices, and stained with a combination of 3% uranyl acetate and lead citrate. These prepared sections were then subjected to the microscope (HT-7800; Hitachi Medical Corporation, Tokyo, Japan).

### 2.8. Flow Cytometry Assay

To assess cell death, HAECs were treated as indicated, then collected and stained with 5 mg/mL Propidium Iodide (PI) (Beyotime, Shanghai, China. ST512) 24 h after radiation. Ferroptosis was a regulated cell death induced by excessive lipid peroxidation, so we conducted lipid peroxidation measurements in cells [14,15,25]. HAECs were harvested 24 h after radiation and incubated with 2 μM BODIPY 581/591 C11 dye (Invitrogen, D3861) for 30 min. Data were collected by FACScan (BD FACS CantoII, Franklin Lakes, NJ, USA) for 20,000 cells and analyzed by FlowJo (Tree Star Inc., version V10, Ashland, OR, USA).

### 2.9. 4D-LFQ Proteomics

The 4D-LFQ adds a fourth dimension (ion mobility) to traditional three-dimensional data (time, *m*/*z*, and ion intensity). Additionally, the label-free technology minimizes potential biases and interferences caused by external labeling agents, providing more reliable and accurate results. Herein, 4D-LFQ proteomics was conducted to explore the target of radiation-induced vascular endothelial ferroptosis. HAECs were assigned to the control group, Fer-1 group, IR group, and IR+Fer-1 groups. The IR+Fer-1 group was pretreated with 5 μM ferrostatin-1 for 24 h and irradiated with a single dose of 16 Gy. The IR group was pretreated with DMSO for 24 h and then irradiated with a single dose of 16 Gy. The control group was pretreated with DMSO for 24 h and then received sham irradiation. Every group had three biological repeats. Protein extraction and trypsin digestion were conducted, followed by liquid chromatography-tandem mass spectrometry analysis-4D mass spectrometer, as previously described [27].

### 2.10. DDHD2 Knockdown in HAECs

To investigate the role DDHD2 played in endothelial ferroptosis, adenovirus encoding negative control (shNC group), and three different DDHD2-targeted shRNA (sh1–3) were applied to infect HAECs in vitro. Oligonucleotide sequences of shRNA are shown in the supplementary material (Supplementary Table S2). Two days after transfection, the fresh medium with 2 μg/mL puromycin was continuously used to purify the cells. The Western Blot was performed to assess protein levels of the DDHD2 gene in infected cells.

### 2.11. Co-Immunoprecipitation (Co-IP) Assay

The Nrf2 (Gene ID: 4780) plasmids were constructed using pCDNA3.1 vectors with a 6× his at the C-terminus. The DDHD2 (Gene ID: 23259) plasmids were sub-cloned to pCDNA3.1 vectors and were C-terminal tagged with 3× flag. The 293T cells were transfected with plasmids carrying Nrf2-his or DDHD2-flag for 48 h and then were lysed with 1 mL NP-40 lysis buffer (Beyotime, Shanghai, China, P0013F). Then, 80 μL lysates were used as input and others were incubated with anti-flag beads (Bimake, Shanghai, China, B26101) on a rotator at 4 °C for 3 h. Immunoprecipitates were centrifuged and washed three times. Then, proteins were boiled and subjected to Western blot analysis.

### 2.12. Statistical Analyses

All data were presented as the mean ± standard error of the mean. Comparisons between multiple groups were analyzed using a one-way ANOVA analysis and Student’s *t*-test was used to compare the two groups. Statistical analyses were conducted utilizing GraphPad Prism 8.0 software. The length and oil-red positive atherosclerotic plaque area were measured with Fiji (http://imagej.nih.gov/ij/ accessed on 8 January 2022). The analysis and visualization of 4D-LFQ proteomics were performed using R (4.2.1). Proteins were annotated using two widely accepted, open-source databases: the Reactome Pathway database (https://reactome.org/ accessed on 29 May 2023) and the Kyoto Encyclopedia of Genes and Genomes (KEGG) database (https://www.genome.jp/kegg/ accessed on 27 May 2023). *p* value < 0.05 is defined as statistically significant.

## 3. Results

### 3.1. Ferroptosis Inhibitors Alleviated Plaque Burdens of RAA Mice

To investigate the baseline plaque burden of *ApoE*−/− mice before radiation, we compared a group subjected to partial ligation of the left common carotid artery (PCL) with the RAA (PCL+IR) group that underwent both PCL and irradiation. The results revealed a significant increase in both length and area of plaques in the RAA group than the PCL group (*p* = 0.004) (Supplementary Figure S1A). Masson’s trichrome staining further demonstrated a reduction in collagen content in plaques of the RAA group than the PCL group. These findings suggested that irradiation could increase plaque burdens and vulnerability of atherosclerotic lesions in *ApoE*−/− mice, and the combination of irradiation with partial ligation of LCCA effectively established the RAA mouse model. 

To explore the role of ferroptosis in RAA, we treated RAA mice with Lip-1, a ferroptosis inhibitor. As results show in Figure 1, the collagen content in the plaques of RAA was lower than in the control group, and this reduction was partially reversed by Lip-1 treatment. This suggests that ferroptosis inhibitors can mitigate the vulnerability of plaques in RAA mice. Furthermore, the oil red staining area (*p* < 0.0001) and lengths (*p* < 0.0001) of the RAA group were significantly increased than those in the control group. However, the RAA+lip-1 group exhibited a significant decrease in both oil red staining area (*p* < 0.0001) and lengths (*p* < 0.0001) of atherosclerosis lesions compared to the RAA group, indicating the increase in plaque burdens in RAA could be alleviated by Lip-1 treatment (Figure 1C–E).

### 3.2. Ferroptosis Inhibitors Ameliorate Radiation-Induced Endothelial Injury and Lipid Peroxidation in RAA

**Figure 2:**
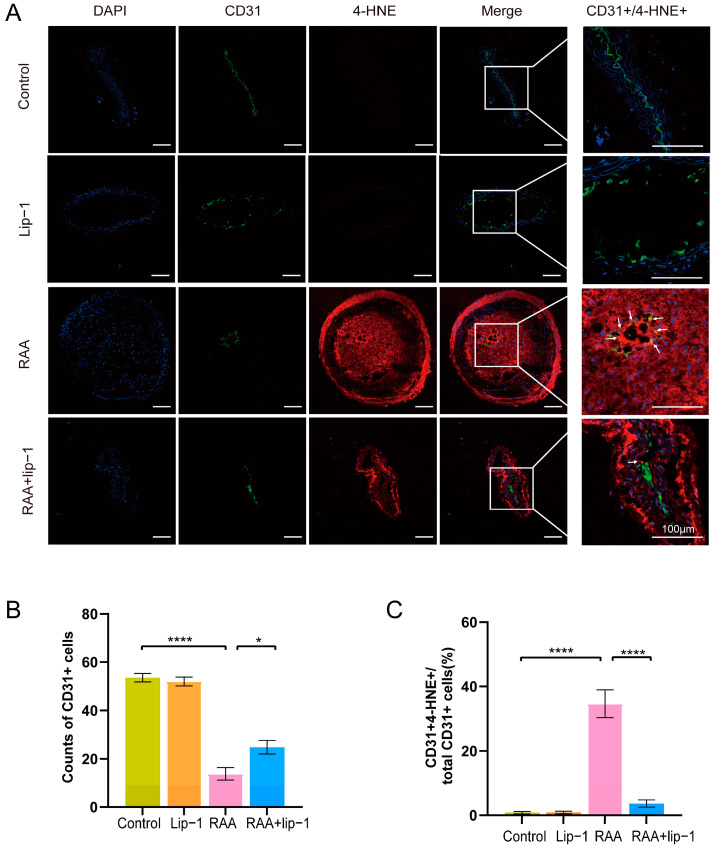
Ferroptosis inhibitor ameliorates lipid peroxidation and endothelial injury of RAA in *ApoE*−/− mice. (**A**) Representative immunofluorescence staining of CD31 (green), 4-HNE (red), and their colocalization (yellow) in carotid atherosclerotic plaques of *ApoE*−/− mice from each group; the arrows depict the yellow cells/double positive cells. (**B**) Quantitative analysis of the counts of CD31+ cells in each group (*n* = 7). (**C**) Quantitative analysis of the co-expression of CD31+/4-HNE+ cells ratio in each group (*n* = 7). * *p* < 0.05; **** *p* < 0.0001.

Endothelial cell injury acts as the trigger in the initiation and progression of RAA. Here, we performed immunofluorescence with endothelial cells marker CD31 and a lipid peroxidation metabolic product 4-HNE. Our results revealed a decrease in the number of CD31+ cells in the RAA group compared to the PCL group, while the co-expression of CD31+/4-HNE+ cells was increased in the RAA group, indicating a reduction in cell numbers and elevated lipid peroxidation in irradiated endothelial cells (Supplementary Figure S1B). Then, we investigated the involvement of ferroptosis in radiation-induced endothelial injury in RAA. Interestingly, we observed a decrease in CD31+ cells in the RAA group compared to the control group, accompanied by an increase in CD31+/4-HNE+ cells in the RAA group (Figure 2A–C). However, after Lip-1 treatment, the number of CD31+ cells in the RAA+lip-1 group increased compared to the RAA group, and the co-positive CD31+/4-HNE+ cells decreased in the RAA+lip-1 group compared to the RAA group, which suggest that radiation may induce ferroptosis to promote endothelial injury in RAA (Figure 2A–C).

### 3.3. Irradiation-Induced Ferroptosis in HAECs

**Figure 3:**
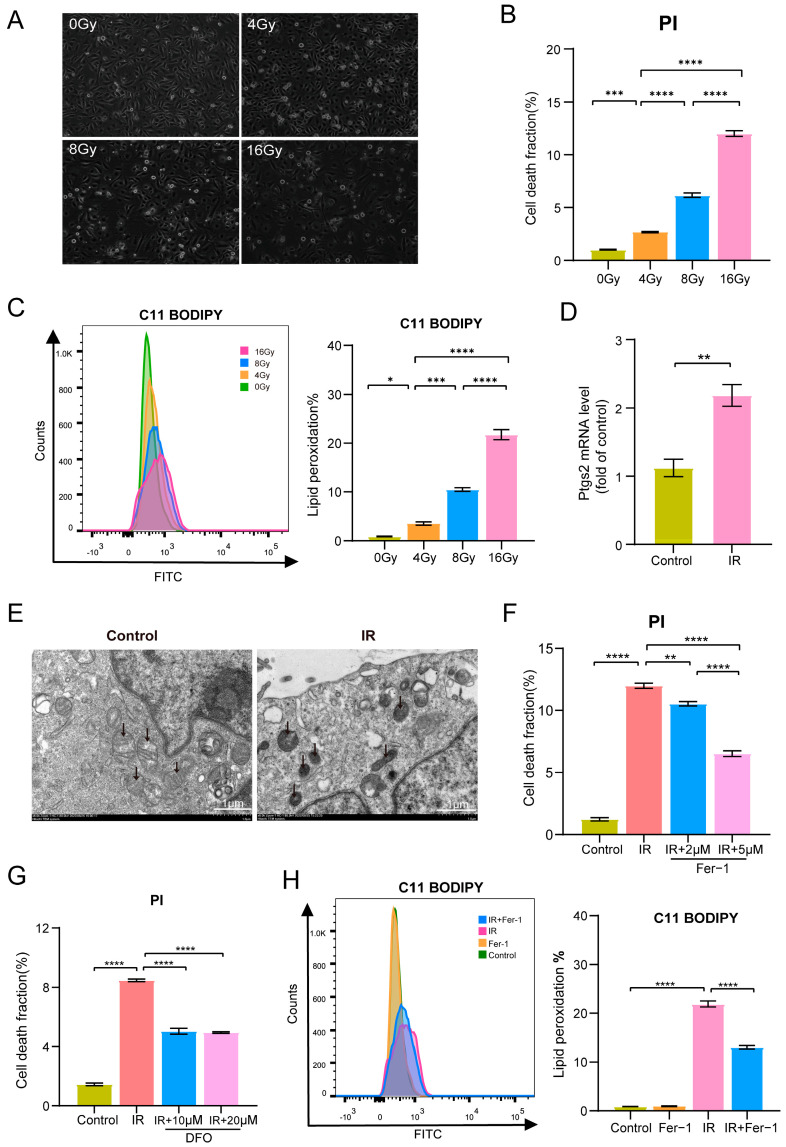
Irradiation promotes HAECs’ death with increased lipid peroxidation. (**A**) Cell morphology of HAECs 24 h after ionizing radiation (IR). Images were taken at an objective magnification of ×20. (**B**) Quantity of cell death fraction of HAECs 24 h after irradiation (*n* = 3) by PI staining. (**C**) Lipid peroxidation assessment in HAECs irradiated with 0, 4, 8, and 16 Gy after 24 h by C11-BODIPY staining (*n* = 3). (**D**) Expression of mRNA PTGS2 in HAECs irradiated with 0 Gy and 16 Gy after 24 h (*n* = 3). (**E**) Transmission electron microscopy images showed that HAECs irradiated with 16 Gy (IR) had shrunken mitochondria with enhanced membrane density at 24 h. Black arrow: mitochondria. (**F**,**G**) Quantity of cell death fraction of HAECs treated with 0 Gy+DMSO (Control), 16 Gy+DMSO (IR), 16 Gy+2–5 μM Fer-1 (IR+Fer-1), and 16 Gy+10–20 μM DFO (IR+DFO) by PI staining (*n* = 3). (**H**) Lipid peroxidation assessment in HAECs treated with 0 Gy+DMSO (control), 0 Gy+Fer-1 (Fer-1), 16 Gy+DMSO (IR), 16 Gy+Fer-1 (IR+Fer-1) after 24 h by C11-BODIPY staining (*n* = 3). * *p* < 0.05; ** *p* < 0.01; *** *p* < 0.001; **** *p* < 0.0001.

Here, we conducted an in vitro model of radiation-induced endothelial injury. We found that the cell death fractions (*p* < 0.0001) (Figure 3A,B) and levels of lipid peroxidation (*p* < 0.0001) (Figure 3C) in HAECs were significantly increased after irradiation with 0, 4, 8, and 16 Gy. According to the above results, we gave the IR group a single dose of 16 Gy in the following experiments and we found that the expression of prostaglandin-endoperoxide synthase 2 (PTGS2) mRNA, a ferroptosis-related gene [28,29], was significantly upregulated in the IR group (*p* = 0.0064) (Figure 3D). 

To further investigate the role of ferroptosis in radiation-induced endothelial injury, we administered ferroptosis inhibitors Fer-1 and an iron ion chelating agent DFO in irradiated HAECs. Transmission electron microscopy revealed that irradiated HAECs exhibited shrunken mitochondria with enhanced membrane density, a characteristic feature of ferroptosis [14] (Figure 3E). Flow cytometry analysis of Propidium Iodide (PI) staining revealed that the IR group exhibited higher cell death fractions compared to the control group (*p* < 0.0001), while the IR+2 μM Fer-1 group (*p* = 0.0029) and IR+5 μM Fer-1 group (*p* < 0.0001) demonstrated lower cell death fractions than the IR group (Figure 3F). Pretreatment of 10 μM DFO (*p* < 0.0001) and 20 μM DFO (*p* < 0.0001) also significantly reduced cell death fractions of irradiated HAECs (Figure 3G). Additionally, Fer-1 partially ameliorated the accumulation of lipid peroxidation induced by irradiation in HAECs (*p* < 0.0001) (Figure 3H). These results suggested that endothelial cell death induced by irradiation was dependent on iron with lipid peroxidation accumulation, indicating that irradiation could induce ferroptosis in HAECs.

### 3.4. Ferroptosis Inhibitor Alleviates Inflammation Cytokines after Irradiation

**Figure 4:**
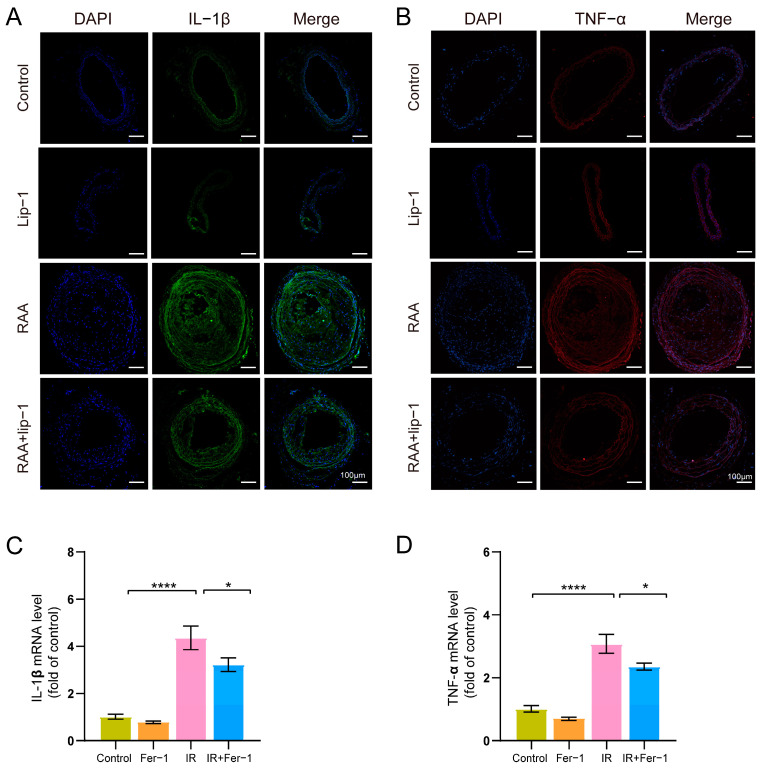
Ferroptosis inhibitor downregulated IL-1β and TNF-α in vivo and in vitro. (**A**) Representative images of immunofluorescence staining of IL-β (green) in carotid atherosclerotic plaques of *ApoE*−/− mice. (**B**) Representative images of immunofluorescence staining of TNF-α (red) in carotid atherosclerotic plaques of *ApoE*−/− mice. (**C**,**D**) Expressions of mRNA IL-1β and TNF-α in HAECs with or without IR and 5 μM Fer-1 treatment at 24 h (*n* = 4). * *p* < 0.05; **** *p* < 0.0001.

Compared to the PCL group, we found that the RAA (PCL+IR) group exhibited upregulated expression of interleukin-1β (IL-1β) and tumor necrosis factor-α (TNF-α) in plaque (Supplementary Figure S1C,D), indicating that irradiation increased the inflammation response in plaque of RAA. Notably, the results revealed the expression of IL-1β and TNF-α in the RAA group were increased compared to the control group, but were mitigated by Lip-1 treatment (Figure 4A,B). Consistent with the in vivo results, we observed that mRNA expressions of IL-1β (*p* = 0.0003) and TNF-α (*p* = 0.0007) in HAECs were significantly increased in the IR group compared to the control group, while the mRNA expressions of IL-1β (*p* = 0.049) and TNF-α (*p* = 0.046) in the IR+Fer-1 group were markedly reduced compared to the IR group (Figure 4C,D). 

### 3.5. DDHD2 May Be the Target of Irradiation-Induced Endothelial Ferroptosis

**Figure 5:**
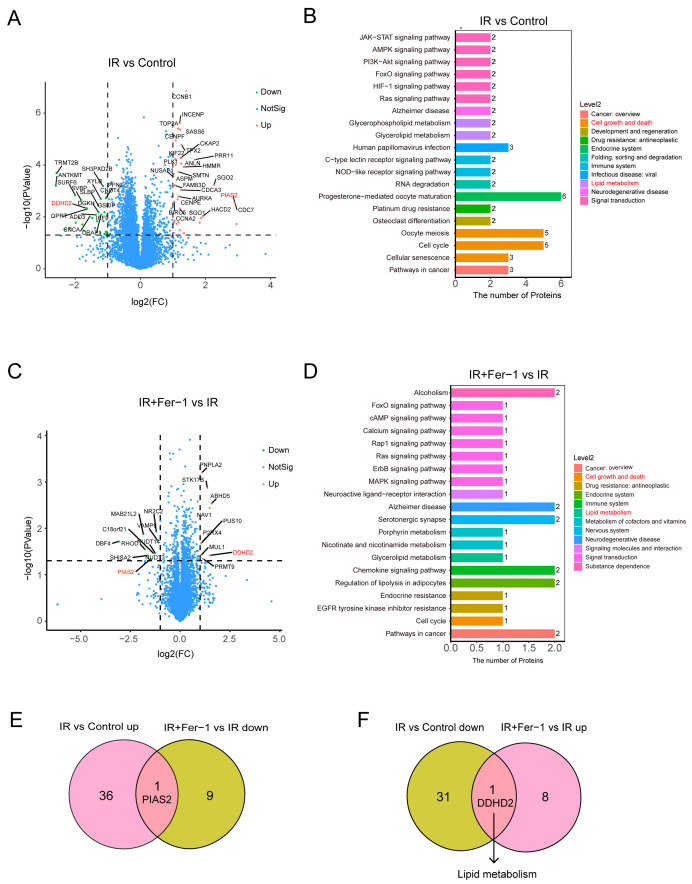
DEPs and KEGG pathway of 4D Label-Free quantitative proteomics analysis. (**A**) Volcano plot showing the DEPs of IR vs. control group (*n* = 3/per group). (**B**) KEGG pathway analysis of DEPs between IR and control group (*n* = 3/per group). (**C**) Volcano plot showing the DEPs of IR+Fer-1 vs. IR group (*n* = 3/per group). (**D**) KEGG pathway analysis of DEPs between IR+Fer-1 and IR group (*n* = 3/per group). (**E**) Venn diagram showing overlap of upregulated DEPs in IR vs. control group and downregulated DEPs in IR+Fer-1 vs. IR group. (**F**) Venn diagram showing overlap of downregulated DEPs in IR vs. control group and upregulated DEPs in IR+Fer-1 vs. IR group and the involved Reactome pathway of DDHD2 (https://reactome.org/ accessed on 29 May 2023). DEPs: Proteins whose adjusted *p*-value < 0.05 and |log2Fc|>1.

To further screen key proteins of radiation-induced endothelial ferroptosis, we collected the cell lysates of HAECs in the control group, Fer-1 group, IR group, and IR+Fer-1 group, and then performed 4D-LFQ proteomics. The differentially expressed proteins (DEPs) were defined by the cut-off values (*p* < 0.05, |log2Fc| > 1). There were 37 upregulated DEPs and 32 downregulated DEPs between the IR group vs. control group (Figure 5A, Supplementary Table S3) and the KEGG pathway analysis highlighted the involvement of these proteins in “cell growth and death” and “lipid metabolism” after irradiation (Figure 5B). Nine upregulated DEPs and ten downregulated DEPs were identified between the IR+Fer-1 group vs. IR group (Figure 5C, Supplementary Table S4). The KEGG pathway analysis showed that some DEPs were also enriched for “cell growth and death” and “lipid metabolism” (Figure 5D). 

**Figure 6:**
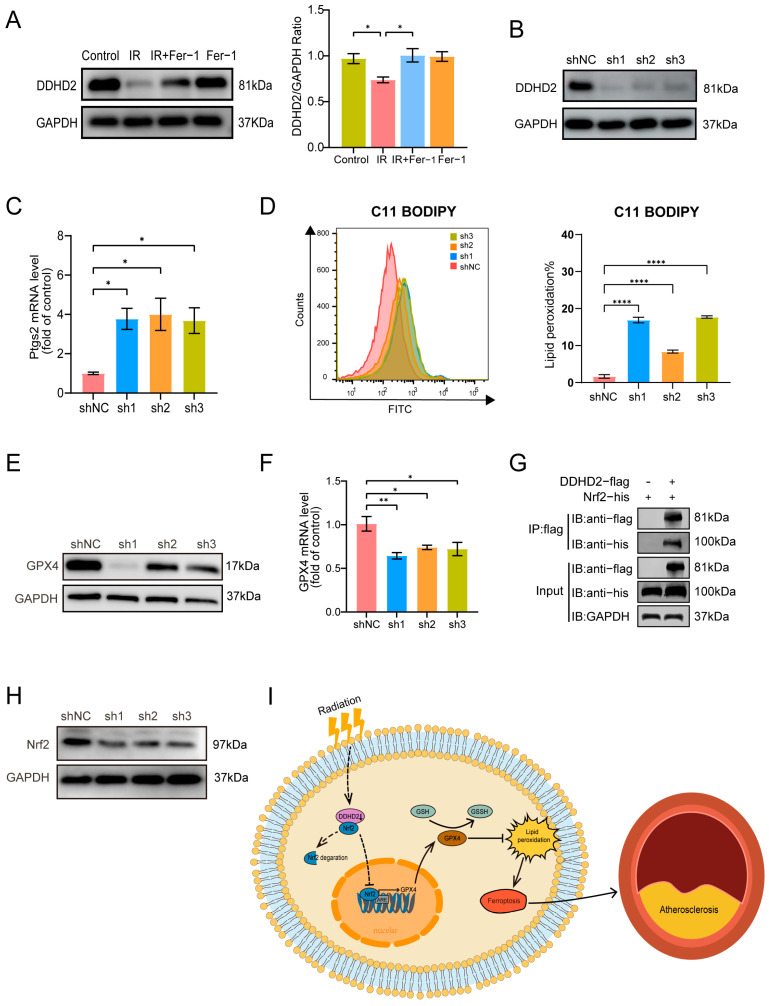
DDHD2 may be the target of irradiation-induced vascular endothelial ferroptosis. (**A**) DDHD2 and GAPDH protein levels in HAECs of each group were evaluated by Western blots (*n* = 3). (**B**) Western blotting analysis of DDHD2 and GAPDH in HAECs, infected with Ad vector-carrying negative control (shNC) and three different DDHD2-knockdown short hairpin RNA expression plasmids (sh1–3) (*n* = 4). (**C**) Expressions of PTGS2 mRNA in shNC; sh1–3 groups were assessed by qRT-PCR. (**D**) Lipid peroxidation assessment in HAECs infected with sh1–3 and shNC by C11-BODIPY staining (*n* = 3). (**E**) GPX4 protein levels in HAECs with shNC, sh1–3 groups, were evaluated by Western blots. (**F**) Expressions of GPX4 mRNA in shNC; sh1–3 groups were assessed by qRT-PCR (*n* = 4). (**G**) Western Blot after co-immunoprecipitation was used to verify the binding of DDHD2 with NRF2. (**H**) Nrf2 protein levels in HAECs with shNC, sh1–3 groups, evaluated by Western blots. (**I**) An illustration depicting the role and underlying mechanism of DDHD2 in radiation-induced endothelial ferroptosis: Downregulation of DDHD2 after radiation causes the release of NRF2 from it and promotes NRF2 degradation, inhibiting the NRF2/GPX4 axis and promoting ferroptosis. * *p* < 0.05; ** *p* < 0.01; **** *p* < 0.0001. Original Western blot images are available in Supplementary Materials.

Then, we performed Venn plots and identified two co-DEPs, protein inhibitors of activated STAT2 Gene (PIAS2) and Domain-Containing Protein 2 (DDHD2). PIAS2 was upregulated in the IR vs. control group and downregulated in the IR+Fer-1 vs. IR group (Figure 5E). DDHD2 was downregulated in the IR vs. control group and upregulated in the IR+Fer-1 vs. IR group (Figure 5F). Notably, DDHD2 is a protein involved with lipid metabolism in the Reactome Pathway (https://reactome.org/) and Western blot also verified that the expression of DDHD2 protein was remarkably downregulated in the IR group compared to the control group (*p* = 0.047), while it was upregulated after Fer-1 treatment (*p* = 0.021) (Figure 6A).

### 3.6. DDHD2 Targets the Nrf2/GPX4 Axis

To investigate the role of DDHD2 in regulating endothelial ferroptosis, DDHD2 was knocked down in HAECs by using three different shRNA (sh1–3) and verified by Western blots (Figure 6B). Our results showed that the expression of PTGS2 mRNA was significantly increased in the sh1 (*p* = 0.026), sh2 (*p* = 0.018), and sh3 (*p* = 0.031) groups when compared to the negative control (shNC) group (Figure 6C), respectively. In addition, a significantly elevated level of lipid peroxidation was detected in the sh1–3 groups compared to the shNC group (*p* < 0.0001) (Figure 6D). Next, we investigated the protein levels of glutathione peroxidase 4 (GPX4), a key enzyme involved in downregulating ferroptosis [29], in DDHD2-knockdown HAECs. Our results revealed a decrease in GPX4 protein expression in sh1–3 groups compared to the control group, along with a reduction in GPX4 mRNA levels (Figure 6E,F). Given that the nuclear factor erythroid 2-like 2 (Nrf2) is an upstream transcription factor of GPX4, we further explored potential interactions between DDHD2 and Nrf2. Through co-immunoprecipitation experiments in 293T cells overexpressing DDHD2-flag and NRF2-his, using anti-flag magnetic beads, we detected the presence of Nrf2-his protein (Figure 6G), indicating an interaction between DDHD2 and Nrf2. Furthermore, the subsequent Western blot analysis revealed that the expression of Nrf2 protein was downregulated in DDHD2-knockdown HAECs (Figure 6H). These results demonstrated the potential regulatory role of DDHD2 in endothelial ferroptosis via the Nrf2/GPX4 pathway.

## 4. Discussion

Previous clinical evidence revealed that radiotherapy for head and neck cancer is associated with increased atherosclerosis, consequently raising the incidence of stroke [6,26,30]. Radiation-induced endothelial injury is recognized as the fundamental trigger and initial phase of atherosclerotic plaque formation and inflammation in RAA [31,32,33]. Therefore, investigating the mechanisms behind radiation-induced endothelial injury is crucial for treating RAA. In this study, we conducted a mouse model with RAA and an in vitro model of irradiated HAECs to validate that radiation-induced endothelial ferroptosis promoted plaque burden and inflammation in RAA. Then, 4D-LFQ proteomics identified DDHD2 as the potential regulatory protein in radiation-induced endothelial ferroptosis. Mechanically, our study further revealed that irradiation could downregulate DDHD2 in endothelial cells to accelerate RAA by inducing ferroptosis through the Nrf2/GPX4 pathway (Figure 6I).

Radiation-induced arterial endothelial ferroptosis is a significant factor in the development of RAA. A prior study has reported that ionizing radiation can trigger ferroptosis in pulmonary endothelial cells by activating via Ca^2+^/Calpain/VE-Cadherin signaling [17]. Consistent evidence suggested that endothelial ferroptosis induced by ox-LDL led to endothelial dysfunction and accelerated atherosclerosis [22]. Moreover, Wu et al. [24] demonstrated that the administration of ferrostatin-1 reduced plaque burden in radiation-induced atherosclerosis and decreased the expression of ferroptosis-related genes in irradiated human umbilical vein endothelial cells. Our results further supported the involvement of ferroptosis in RAA, showing that ferroptosis inhibitors decreased radiation-induced endothelial injury, plaque burden and cytokine levels in RAA. Additionally, our in vitro results showed that irradiation induced cell death in HAECs through iron-dependent lipid peroxidation, characterized by mitochondrial shrinkage observed in TEM, which was mitigated by ferroptosis inhibitors. These findings indicated that radiation could induce endothelial ferroptosis to promote endothelial injury and accelerate the development of RAA. 

DDHD2 may be an essential protein of radiation-induced vascular endothelial ferroptosis. DDHD2, also named KIAA0725p, is a lipid-metabolizing enzyme belonging to the intracellular phospholipase A1 family [34]. Nakajima et al. also reported that DDHD2 effectively hydrolyzed phospholipids, especially phosphatidic acid and phosphatidylethanolamine, in 293T cells [35]. Patients with DDHD2 mutations had lipid accumulation in the brain and hereditary spastic paraplegia [36]. Ferroptosis is characterized by lipid metabolizes dysfunction and accumulation of lipid peroxidation. Polyunsaturated fatty acids-phospholipids are substrates of ferroptosis and high levels of phospholipids means an increased sensitivity to ferroptosis [37]. The phospholipase A2 family β (iPLA2β) was reported to be a ferroptosis suppressor, which could cleave the oxidized PUFA-hydroperoxides tail from phospholipids to reduce lipid peroxidation [38]. The Reactome pathway (http://reactome.org/ accessed on 29 May 2023) showed that the function and pathway of DDHD2 were similar to iPLA2β and our findings revealed an upregulation of lipid peroxidation in DDHD2-knockdown HAECs. In addition, 4D-LFQ proteomics and Western blots also demonstrated that DDHD2 was significantly downregulated by irradiation and ameliorated by the ferroptosis inhibitor, indicating that DDHD2 may serve as a potential target in radiation-induced vascular endothelial ferroptosis. 

DDHD2 targets the Nrf2/GPX4 axis to regulate ferroptosis. GPX4 is known as an important defender of ferroptosis, which can convert lethal lipid hydroperoxides to nontoxic lipid alcohols relying on glutathione, thereby reducing lipid peroxidation [29]. Nrf2, a key transcription regulator, maintains cellular redox homeostasis by regulating expressions of multiple antioxidant response element (ARE)-containing genes, such as GPX4 [39]. Lv et al. [40] have reported that activation of the Nrf2/GPX4 pathway alleviated endothelial cell ferroptosis in postmenopausal atherosclerosis. A similar study by Zhao et al. [41] confirmed that bicyclol could prevent ferroptosis by upregulating the Nrf2/GPX4 axis in acute liver injury mice. A previous study also showed that proteins could regulate the transcription of GPX4 by interacting with Nrf2 [42]. Interestingly, in our study, decreased expression of GPX4 in protein and mRNA levels were also observed in HAECs with DDHD2 knockdown. Meanwhile, Co-IP showed that DDHD2 interacted with Nrf2, an upstream transcript factor of GPX4. Nrf2 tends to form a complex with Kelch-like ECH-associated protein 1 (Keap1) and is degraded by the ubiquitination-proteasome signaling pathway [43]. A study by Xu et al. [44] identified a protein capable of antagonizing Keap1, thereby preventing the degradation of Nrf2. Notably, our study also observed the downregulation of Nrf2 protein in DDHD2-knockdown HAECs, indicating that Nrf2 degradation occurred following its release from DDHD2. Thus, we proposed that radiation induced vascular endothelial ferroptosis by downregulating DDHD2, and DDHD2 may be a ferroptosis suppressor through the Nrf2/GPX4 pathway. 

This study also presented some limitations. Firstly, our animal model was treated with a combination of PCL, and irradiation, in which irradiation increased the risk of atherosclerosis but was not the sole contributing factor. The irradiated HAECs model partially addresses this limitation by focusing on the direct effect of irradiation on targeted cells. Secondly, how ionizing radiation downregulates DDHD2 in HAECs remains unclear in our study, and a further mechanism is needed to evaluate in vivo and in vitro. 

## Data Availability

The data generated in this study are available within the article and its Supplementary Data Files. The 4D-LFQ proteomics data in this study are available upon reasonable request from the corresponding author.

## Supplementary Materials

The following supporting information can be downloaded at: https://www.mdpi.com/article/10.3390/biom14070879/s1, Figure S1: Radiation accelerated atherosclerosis. (A) Representative images of microscopy and sections stained with hematoxylin and eosin, Masson, and Oil Red of the left carotid artery. (B) Representative immunofluorescence staining of CD31 (green), 4-HNE (red), and their colocalization (yellow) in carotid atherosclerotic plaques of *ApoE*−/− mice, the arrows depict the yellow cells/double positive cells. (C,D) Representative images of immunofluorescence staining of IL-1β (green) and TNF-α (red) in carotid atherosclerotic plaques of *ApoE*−/− mice. * *p* < 0.05; ** *p* < 0.01; Table S1: The list of primers used in qRT-PCR; Table S2: Oligonucleotide sequences of shRNA; Table S3: Differential expression proteins of IR vs. control in HAECs. Table S4: Differential expression proteins of IR+Fer-1 vs. IR in HAECs.
