# Supplementary material for: Radiation-Induced Endothelial Ferroptosis Accelerates Atherosclerosis via the DDHD2-Mediated Nrf2/GPX4 Pathway
Source: Biomolecules. 2024 Jul 22;14(7):879. doi: 10.3390/biom14070879 (PMC11274403; doi:10.3390/biom14070879)
Supplement: Supplementary file 1 [file biomolecules-14-00879-s001.zip › supplementary files/Figure S1.pdf]

Figure S1

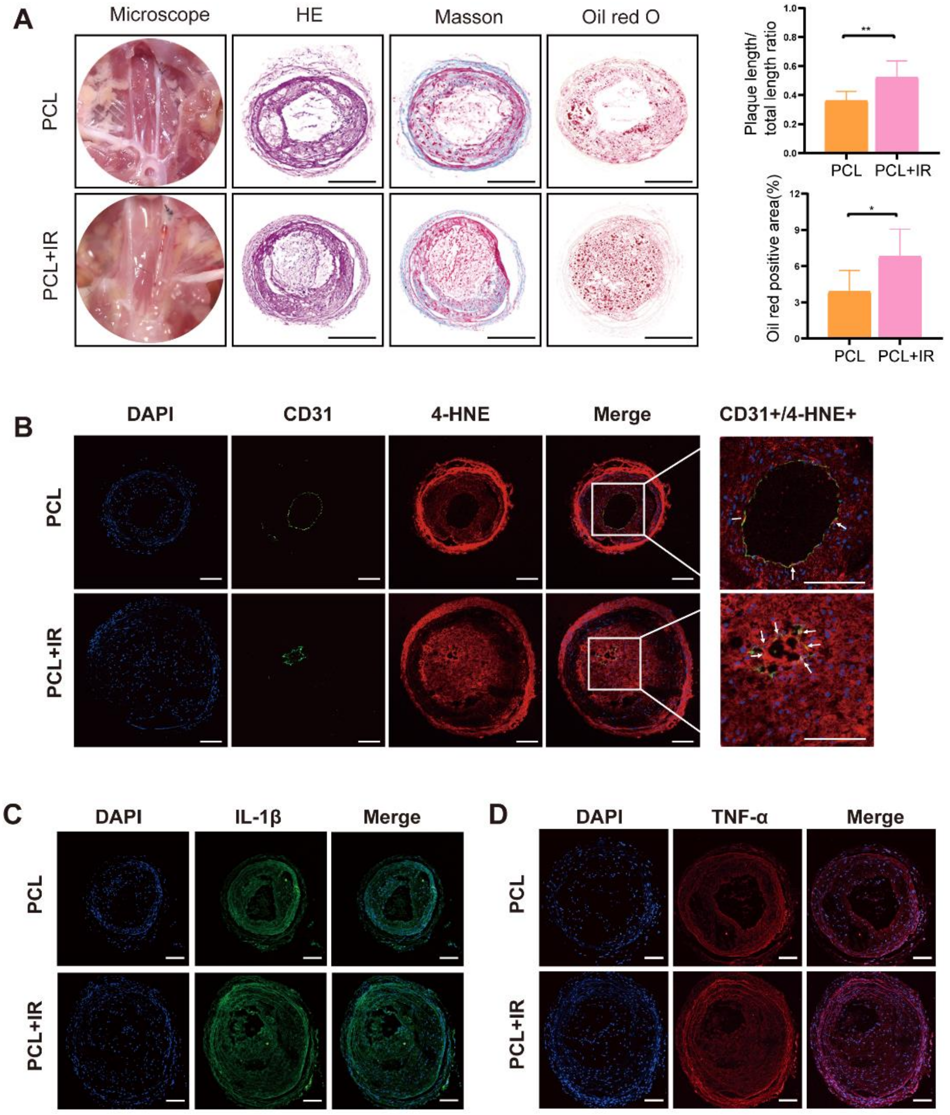

Figure S1. Radiation accelerated atherosclerosis. (A) Representative images of microscopy and sections stained with hematoxylin and eosin, Masson, and Oil Red of the left carotid artery. (B) Representative immunofluorescence staining of CD31 (green), 4-HNE (red), and their colocalization (yellow) in carotid atherosclerotic plaques of *ApoE*$^{-/-}$ mice, the arrows depict the yellow cells/double positive cells. (C, D) Representative images of immunofluorescence staining of IL-1β (green) and TNF-α (red) in carotid atherosclerotic plaques of *ApoE*$^{-/-}$ mice. *p<0.05; **p<0.01; ***p<0.001; ****p<0.0001.
