# Supplementary material for: Radiation-Induced Endothelial Ferroptosis Accelerates Atherosclerosis via the DDHD2-Mediated Nrf2/GPX4 Pathway
Source: Biomolecules. 2024 Jul 22;14(7):879. doi: 10.3390/biom14070879 (PMC11274403; doi:10.3390/biom14070879)
Supplement: Supplementary file 1 [file biomolecules-14-00879-s001.zip › supplementary files/Table S1.pdf]

Table S1. The list of primers used in qRT-PCR.

Gene name	Primers
β-actin Forward	TGTACCCAGGCATTGCTGAC
β-actin Reverse	AACGCAGCTCAGTAACAGTCC
PTGS2 Forward	CTGCGCCTTTTCAAGGATGG
PTGS2 Reverse	GGGGATACACCTCTCCACCA
TNF-α Forward	CAGGCGGTGCCTATGTCTC
TNF-α Reverse	CGATCACCCCGAAGTTCAGTAG
IL-1β Forward	TGCCACCTTTTGACAGTGATG
IL-1β Reverse	AAGGTCCACGGGAAAGACAC
DDHD2 Forward	CAAGGTCGGCCAAGATCAGT
DDHD2 Reverse	GGTTCAAGGAAACACTTCGAAAATC
GPX4 Forward	TTCCCCTGCAACCAGTTTGG
GPX4 Reverse	ACCACGCAGCCGTTCTTATC
