# Supplementary material for: Radiation-Induced Endothelial Ferroptosis Accelerates Atherosclerosis via the DDHD2-Mediated Nrf2/GPX4 Pathway
Source: Biomolecules. 2024 Jul 22;14(7):879. doi: 10.3390/biom14070879 (PMC11274403; doi:10.3390/biom14070879)
Supplement: Supplementary file 1 [file biomolecules-14-00879-s001.zip › supplementary files/Table S2.pdf]

Table S2. Oligonucleotide sequences of shRNA

Name	shRNA 序列
sh1 Forward	CcggGCAGCTTGTATGAACCAGTTTCTCGAGAAACTGGTTCATACAAGCTGCTTTTTTg
sh1 Reverse	aattcaaaaaaGCAGCTTGTATGAACCAGTTTCTCGAGAAACTGGTTCATACAAGCTGC
sh2 Forward	CcggGCAGCTGGAAGAGGCATATTTCAAGAGAATATGCCTCTTCCAGCTGCTTTTTTg
sh2 Reverse	aattcaaaaaaGCAGCTGGAAGAGGCATATTCTCTTGAAATATGCCTCTTCCAGCTGC
sh3 Forward	CcggGCTGTAACTTTGGATGAATTTCAAGAGAATTCATCCAAAGTTACAGCTTTTTTg
sh3 Reverse	aattcaaaaaaGCTGTAACTTTGGATGAATTCTCTTGAAATTCATCCAAAGTTACAGC
NC Forward	CcggCCTAAGGTTAAGTCGCCCTCGCTCGAGCGAGGGCGACTTAACCTTAGGTTTTTTg
NC Reverse	aattcaaaaaaCCTAAGGTTAAGTCGCCCTCGCTCGAGCGAGGGCGACTTAACCTTAGG
