# Supplementary material for: Radiation-Induced Endothelial Ferroptosis Accelerates Atherosclerosis via the DDHD2-Mediated Nrf2/GPX4 Pathway
Source: Biomolecules. 2024 Jul 22;14(7):879. doi: 10.3390/biom14070879 (PMC11274403; doi:10.3390/biom14070879)
Supplement: Supplementary file 1 [file biomolecules-14-00879-s001.zip › supplementary files/Table S3.pdf]

Table S3. Differential expression proteins of IR vs control in HAECs

Protein ID	Gene Name	IR/control	t test p value
Q96ND0	FAM210A	7.744757170	0.01873056
O00311	CDC7	7.530223649	0.00303743
O75928	PIAS2	7.439369364	0.00282946
Q562F6	SGO2	4.798625383	0.00058812
Q6Y1H2	HACD2	3.800098891	0.00929397
Q9NYP3	DONSON	3.646751035	0.01090470
Q14596	NBR1	3.531592076	0.01663995
Q7LGC8	CHST3	3.244294790	0.04849517
Q96HE9	PRR11	3.185410904	0.00006178
Q5FBB7	SGO1	2.907622163	0.00623781
P14635	CCNB1	2.653908083	0.00000014
Q86UL3	GPAT4	2.532233779	0.04202461
O75330	HMMR	2.520389728	0.00011819
Q9ULW0	TPX2	2.400337759	0.00001696
Q9NQW6	ANLN	2.384266886	0.00008745
Q99618	CDCA3	2.376417301	0.00091335
Q8WWK9	CKAP2	2.360802358	0.00005333
P20248	CCNA2	2.321507443	0.00476372
Q6UVJ0	SASS6	2.317737750	0.00000424
O43196	MSH5	2.315725675	0.03249895
Q9NQS7	INCENP	2.308058463	0.00000245
P53814	SMTN	2.257659741	0.00018212
O14965	AURKA	2.253567924	0.00163235
Q6NSJ0	MYORG	2.250861725	0.01611318
P11388	TOP2A	2.228664507	0.00000392
Q02224	CENPE	2.211753800	0.00212041
Q96LT9	RNPC3	2.162015188	0.01842176
P49454	CENPF	2.120360197	0.00001123
P53350	PLK1	2.118051014	0.00009740
Q14807	KIF22	2.111384105	0.00005801
Q9H4H8	FAM83D	2.081465060	0.00060141
P49184	DNASE1L1	2.075030320	0.01410964
O15392	BIRC5	2.055722133	0.00254429
Q9ULL5	PRR12	2.042898020	0.01616809
P82921	MRPS21	2.038517978	0.04088925
Q8IZT6	ASPM	2.032637931	0.00022486
Q9BXS6	NUSAP1	2.003540920	0.00028320
Q13541	EIF4EBP1	0.491817364	0.03834482
Q00978	IRF9	0.490335139	0.00876695
P49914	MTHFS	0.485084256	0.04651798
P60673	PFN3	0.481487580	0.00187099
O43310	CTIF	0.481044082	0.03701277
O95628	CNOT4	0.479641578	0.00121262
P16930	FAH	0.467028909	0.04498072
Q9P0R6	GSKIP	0.466409438	0.00652807
A2RUC4	TYW5	0.456821812	0.01436671
A1X283	SH3PXD2B	0.451701247	0.00065054

Q14919	DRAP1	0.433998286	0.00826571
O75191	XYLB	0.431635544	0.00203048
Q9NQ86	TRIM36	0.426342376	0.04157761
Q86XP1	DGKH	0.412151404	0.00664772
Q14493	SLBP	0.408300591	0.00360520
Q9UMZ2	SYNRG	0.399422820	0.01463183
Q9UEY8	ADD3	0.386741335	0.00830520
Q9Y3Q8	TSC22D4	0.381880075	0.02663856
Q9UKT4	FBXO5	0.359887038	0.01103248
P37840	SNCA	0.340516628	0.00976053
Q15274	QPRT	0.320267460	0.00494439
O94830	DDHD2	0.320032445	0.00467350
Q9BQ15	NABP2	0.302869871	0.03363951
P13929	ENO3	0.291885026	0.01945215
Q8N300	SVBP	0.283643859	0.00224873
B3EWG6	FAM25G	0.271111610	0.01037637
O15391	YY2	0.252940249	0.01663742
O75683	SURF6	0.250198193	0.00145362
Q9NZC9	SMARCAL1	0.223192195	0.02619820
O75223	GGCT	0.198756687	0.03046323
Q96GJ1	TRMT2B	0.169537233	0.00020471
Q9BQD7	ANTKMT	0.166606560	0.00062645
