# Supplementary material for: Radiation-Induced Endothelial Ferroptosis Accelerates Atherosclerosis via the DDHD2-Mediated Nrf2/GPX4 Pathway
Source: Biomolecules. 2024 Jul 22;14(7):879. doi: 10.3390/biom14070879 (PMC11274403; doi:10.3390/biom14070879)
Supplement: Supplementary file 1 [file biomolecules-14-00879-s001.zip › supplementary files/Table S4.pdf]

Table S4. Differential expression proteins of IR+Fer-1 vs IR

Protein	Gene Name	IR+Fer-1/IR	t test p value
Q8WTS1	ABHD5	2.963379059	0.00292918
O94830	DDHD2	2.809985128	0.038248397
Q8NEY1	NAV1	2.788980273	0.003657526
O94768	STK17B	2.56561943	0.001342238
Q99571	P2RX4	2.249719164	0.018238028
Q6P2P2	PRMT9	2.239030554	0.045911741
Q96AD5	PNPLA2	2.139249966	0.000626378
Q3MIT2	PUS10	2.084754406	0.020890641
Q969V5	MUL1	2.083363083	0.03761763
O95989	NUDT3	0.482775529	0.049564971
Q9BV40	VAMP8	0.452447733	0.018023656
P49116	NR2C2	0.435943324	0.011725158
O95848	NUDT14	0.41829362	0.034157739
Q32NC0	C18orf21	0.392847943	0.034267508
O75928	PIAS2	0.358214943	0.045882634
Q6UWI4	SHISA2	0.32023855	0.038448767
O00212	RHOD	0.293628247	0.028411039
Q9Y586	MAB21L2	0.277530948	0.013341809
Q9UBU7	DBF4	0.11895806	0.01916708
