# Supplementary figures and images for: Radiation-Induced Endothelial Ferroptosis Accelerates Atherosclerosis via the DDHD2-Mediated Nrf2/GPX4 Pathway
Source: Biomolecules. 2024 Jul 22;14(7):879. doi: 10.3390/biom14070879 (PMC11274403; doi:10.3390/biom14070879)

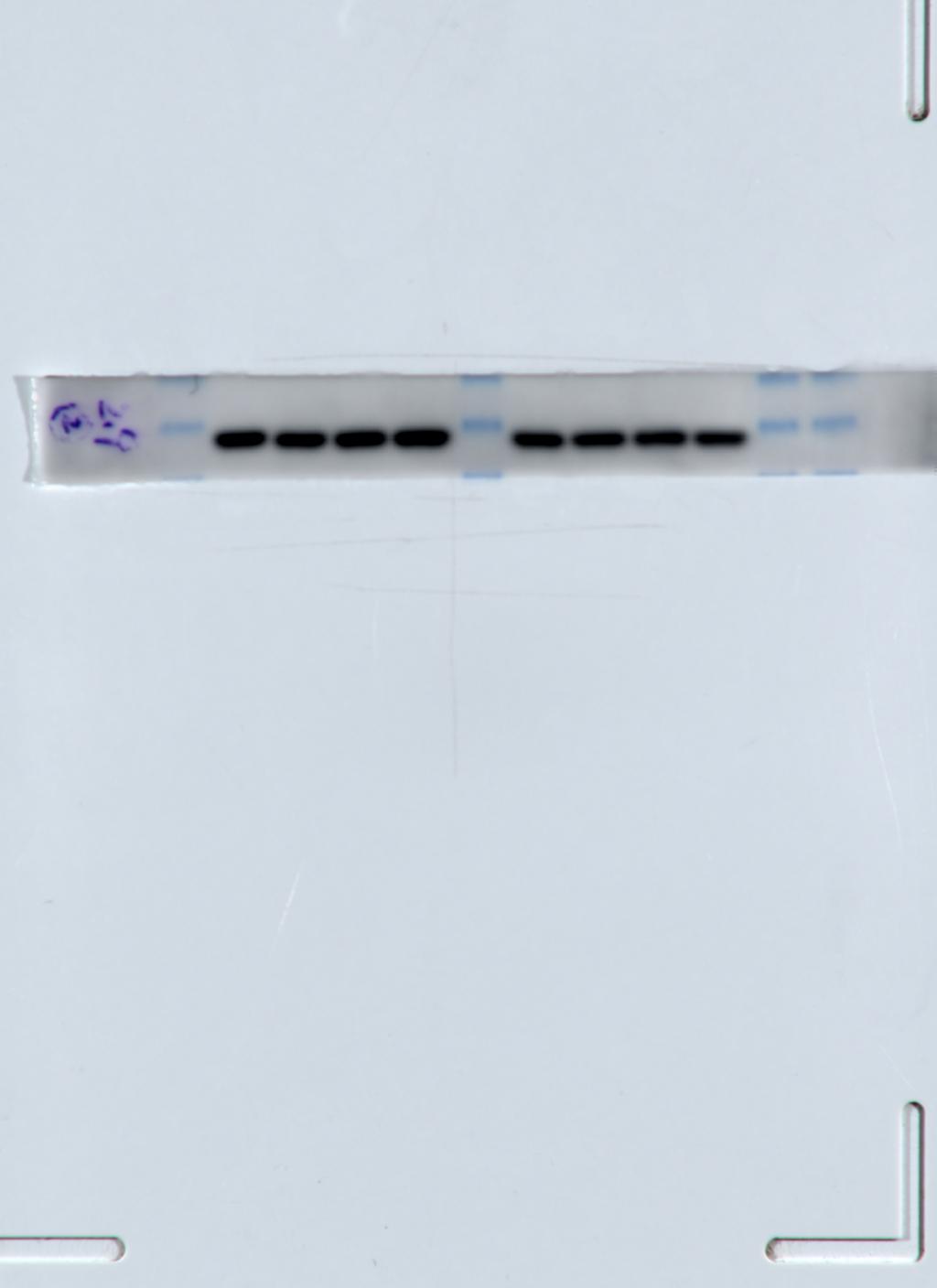

Supplement: Supplementary file 1 [file biomolecules-14-00879-s001.zip › WB_rawdata/Fig6A gapdh.jpg]

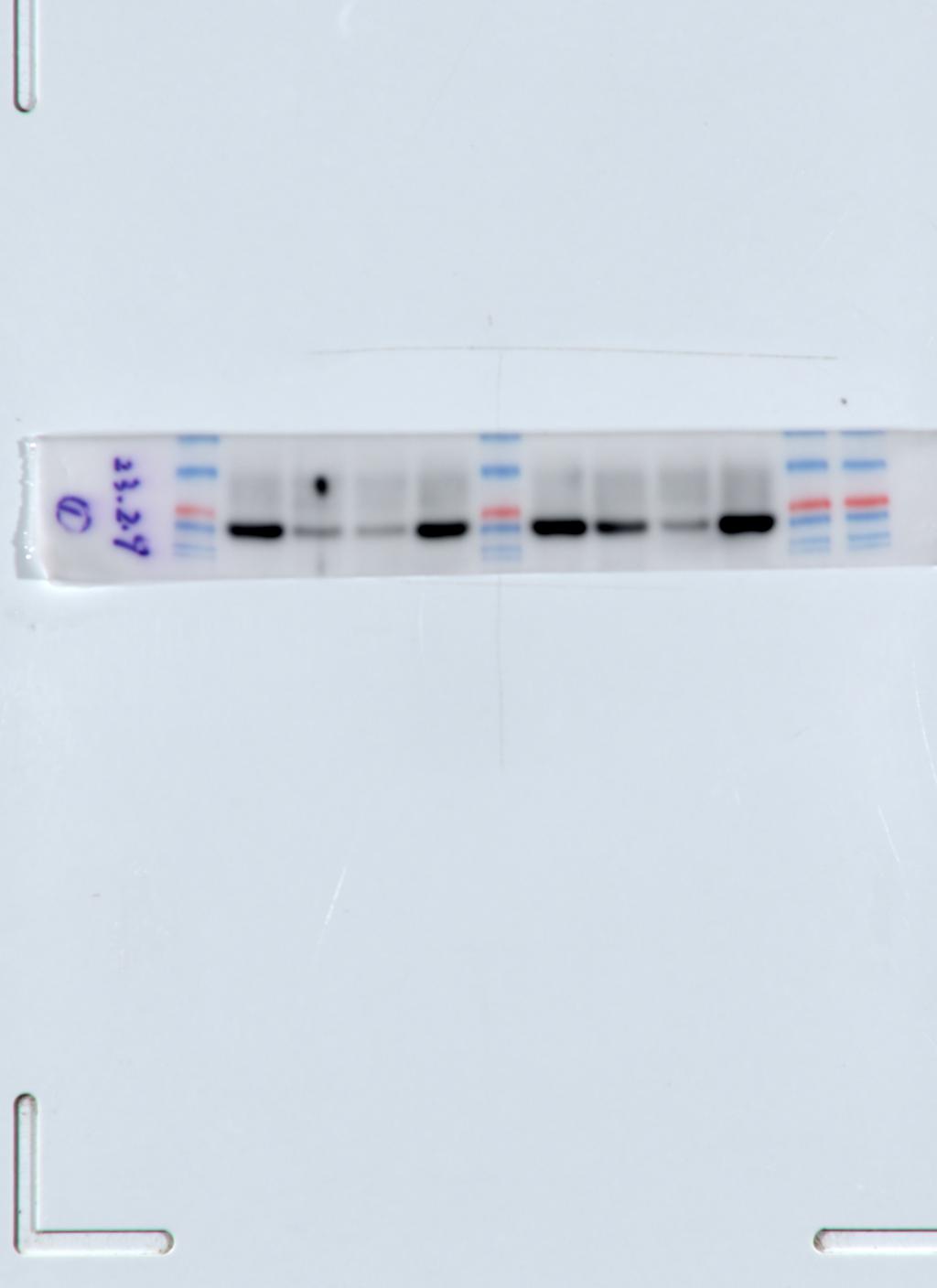

Supplement: Supplementary file 1 [file biomolecules-14-00879-s001.zip › WB_rawdata/Fig6A_DDHD2.jpg]

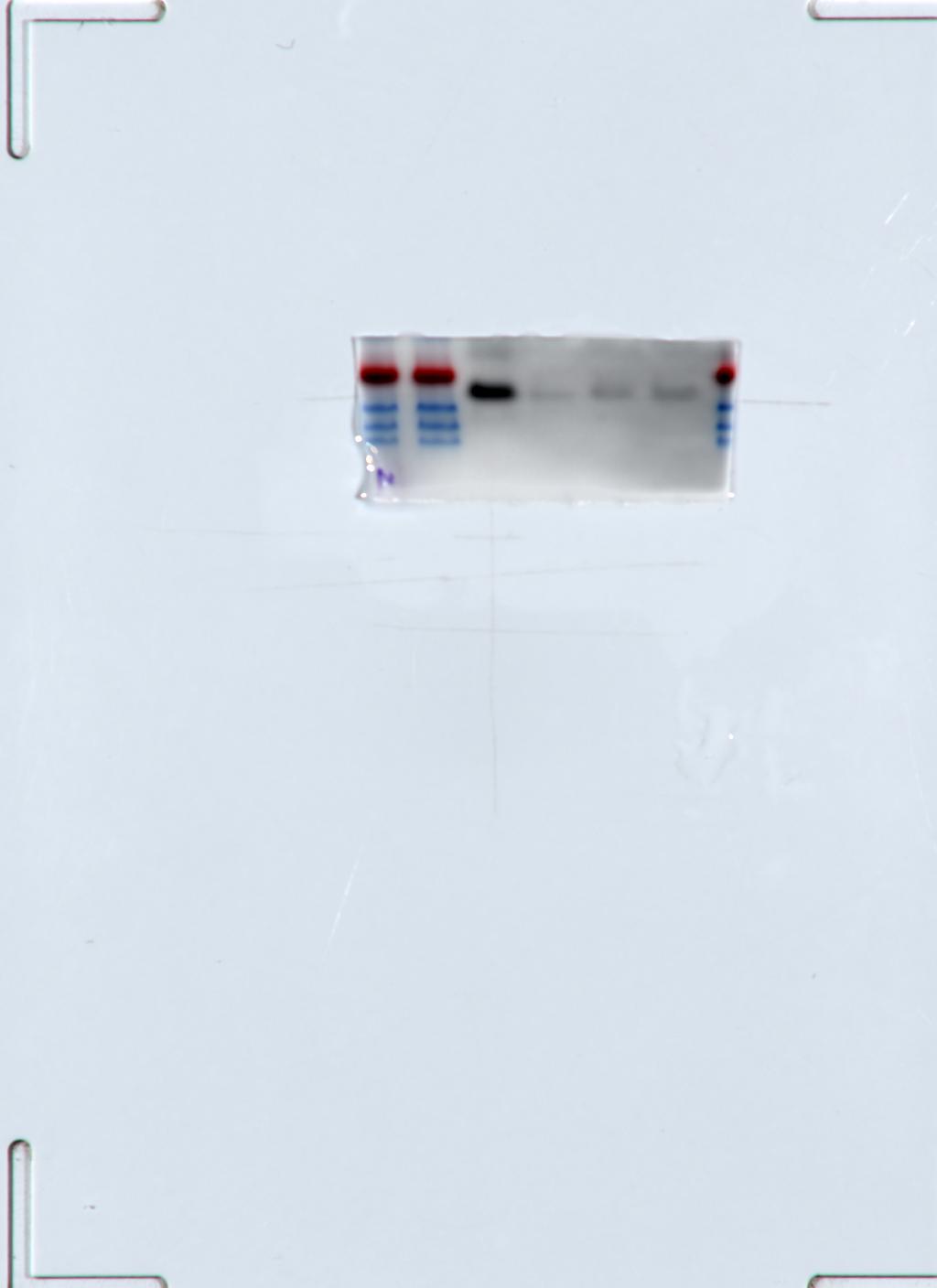

Supplement: Supplementary file 1 [file biomolecules-14-00879-s001.zip › WB_rawdata/Fig6C_ddhd2.jpg]

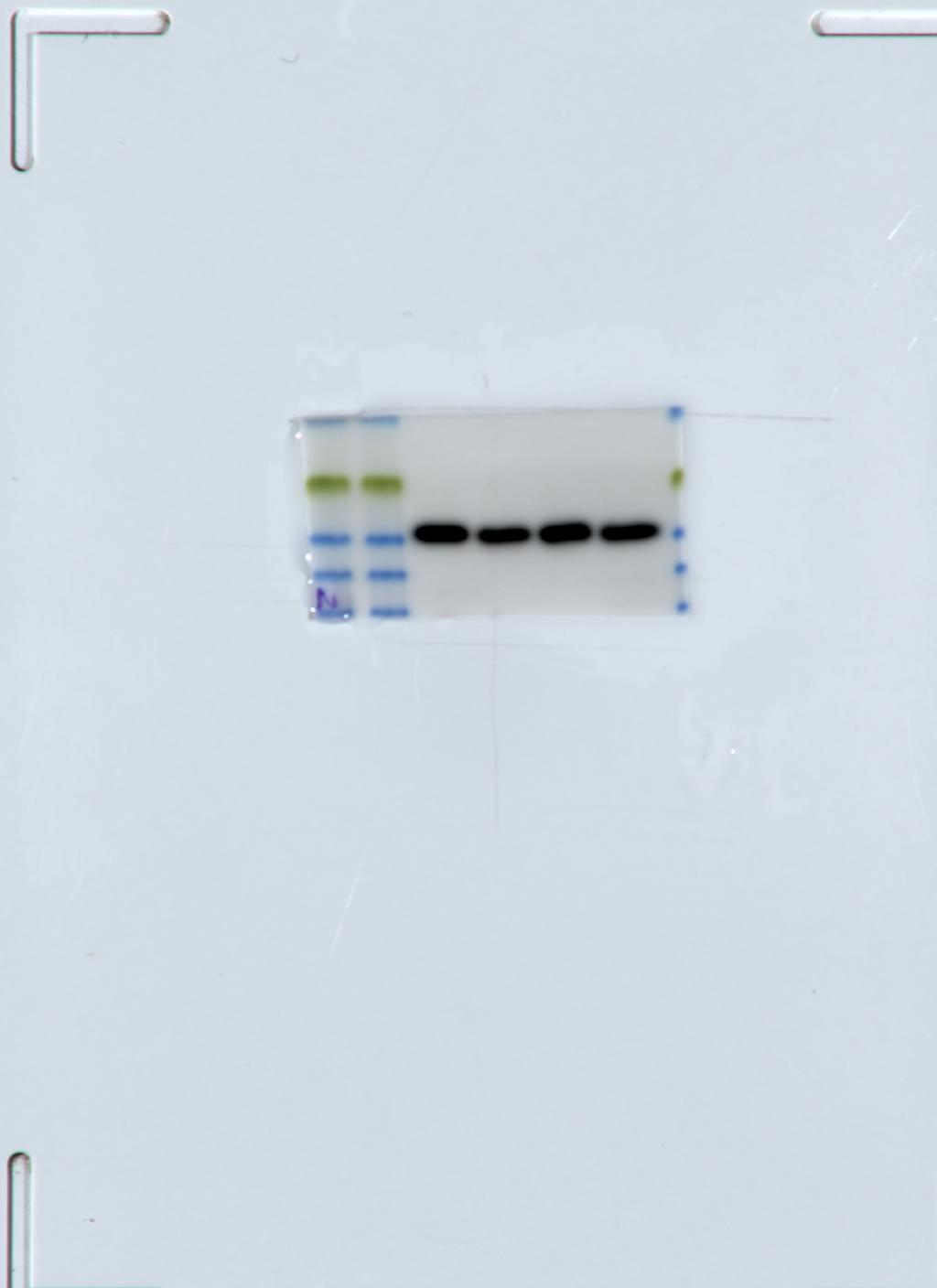

Supplement: Supplementary file 1 [file biomolecules-14-00879-s001.zip › WB_rawdata/Fig6C_gapdh.jpg]

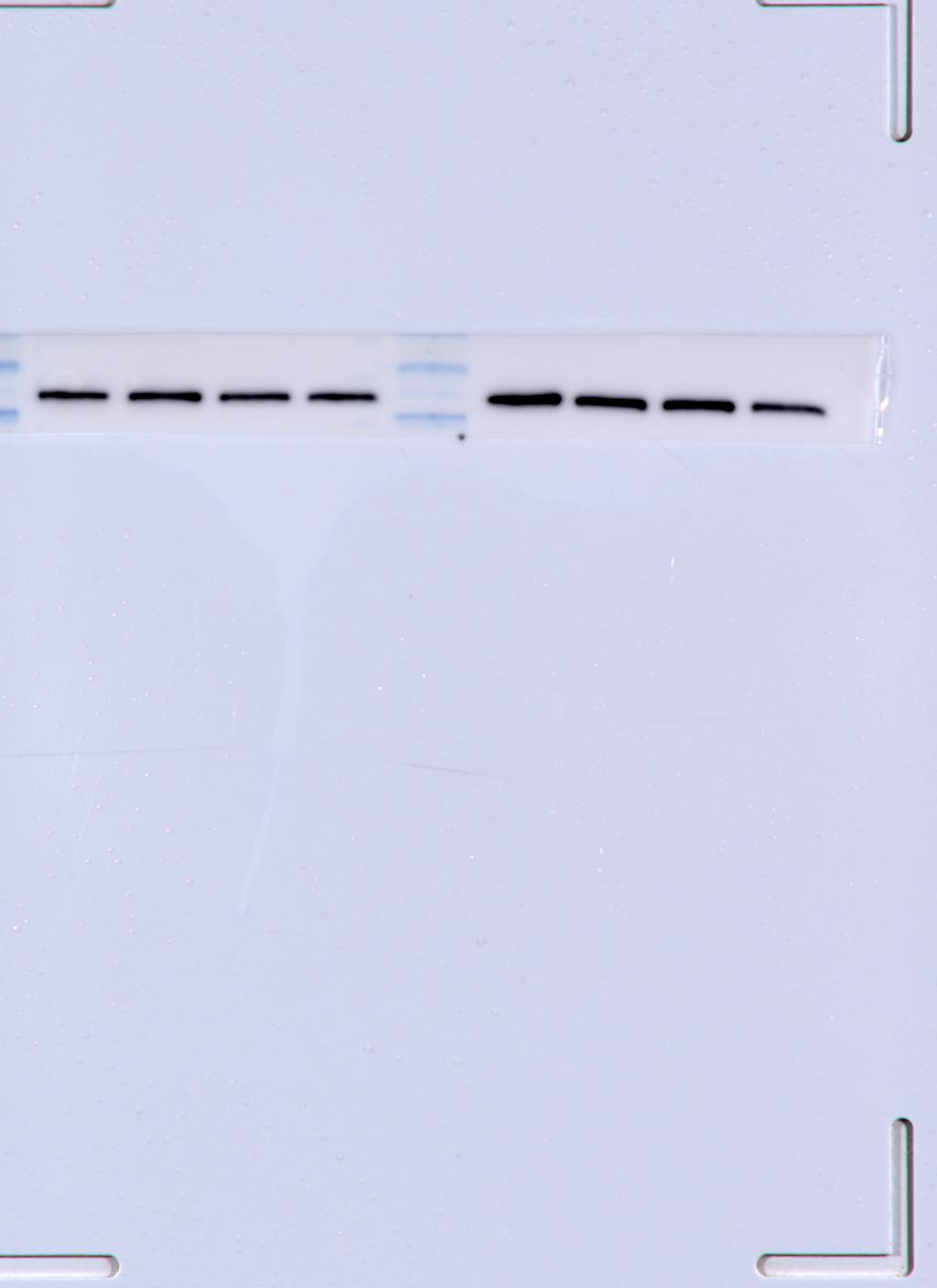

Supplement: Supplementary file 1 [file biomolecules-14-00879-s001.zip › WB_rawdata/Fig6E_gapdh.jpg]

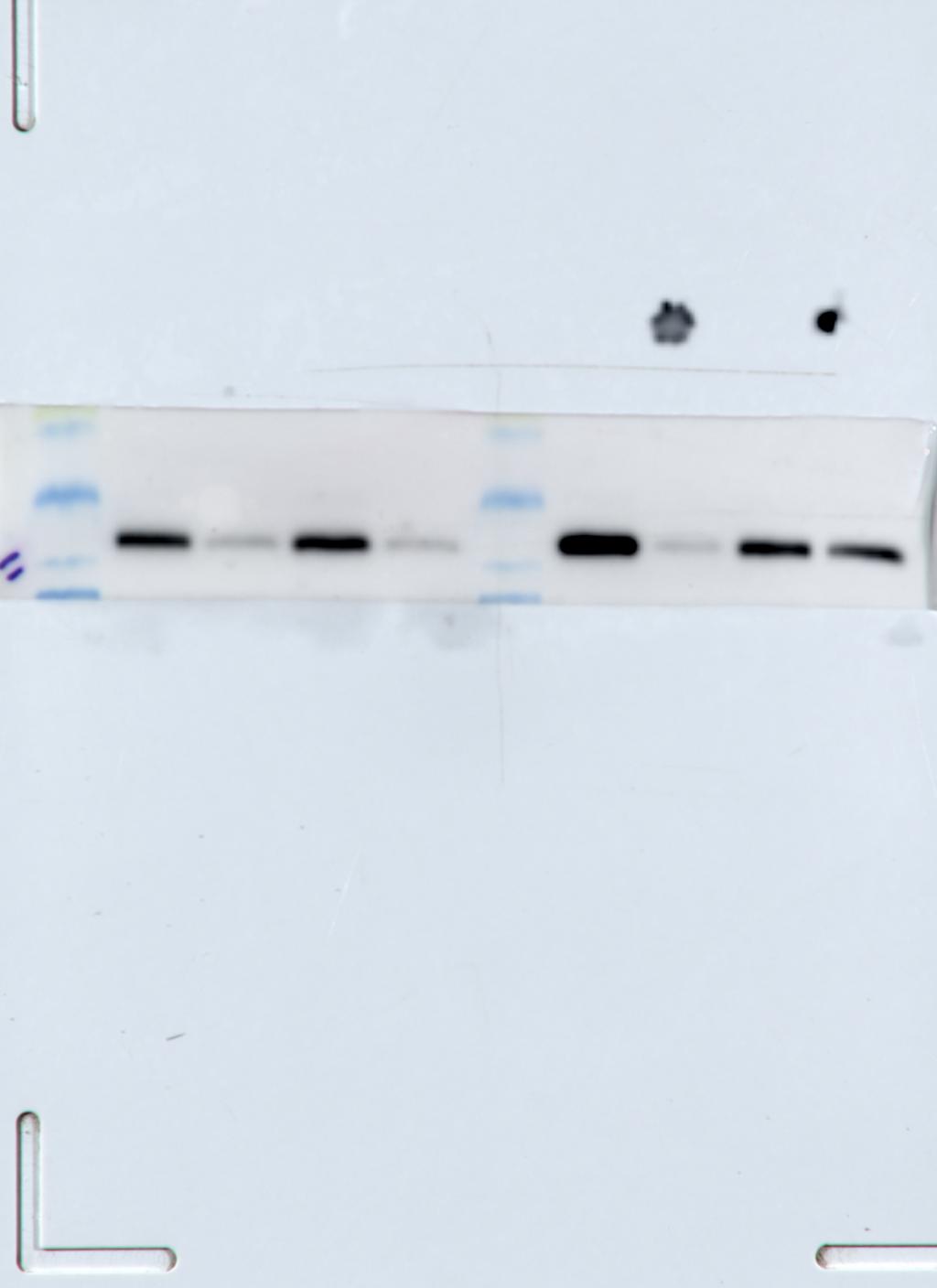

Supplement: Supplementary file 1 [file biomolecules-14-00879-s001.zip › WB_rawdata/Fig6E_gpx4.jpg]

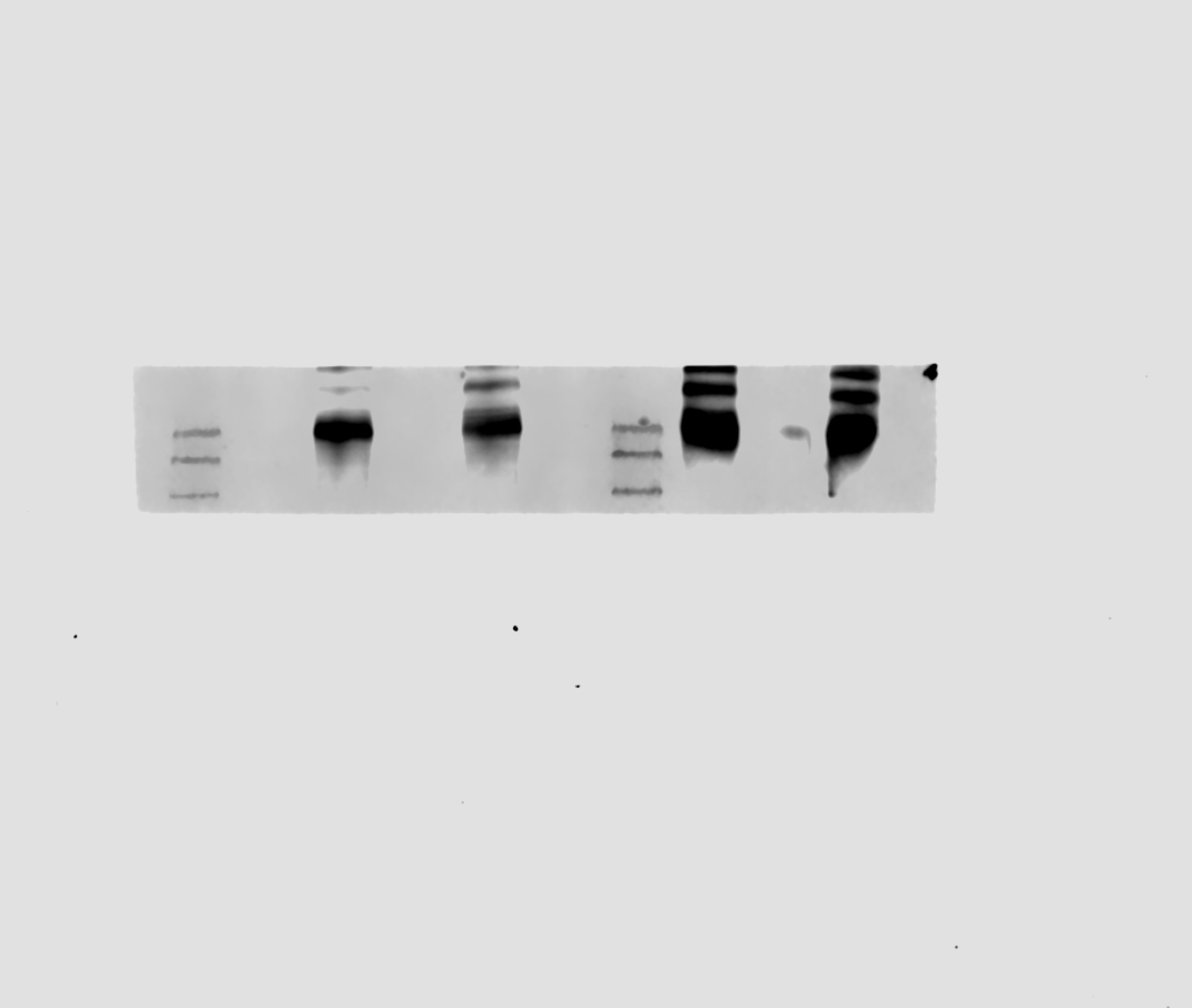

Supplement: Supplementary file 1 [file biomolecules-14-00879-s001.zip › WB_rawdata/Fig6G_DDHD2-flag.tif]

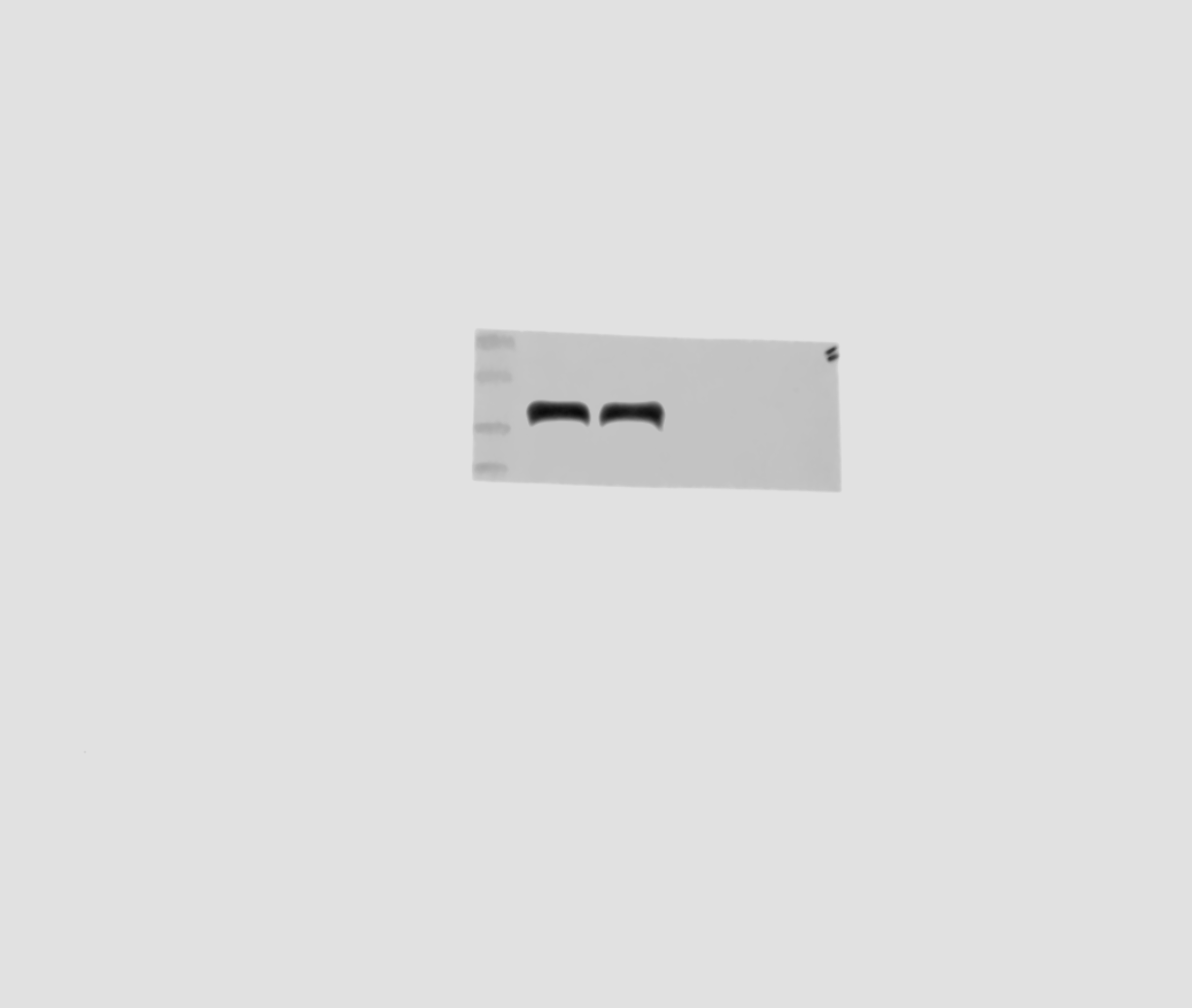

Supplement: Supplementary file 1 [file biomolecules-14-00879-s001.zip › WB_rawdata/Fig6G_gapdh.tif]

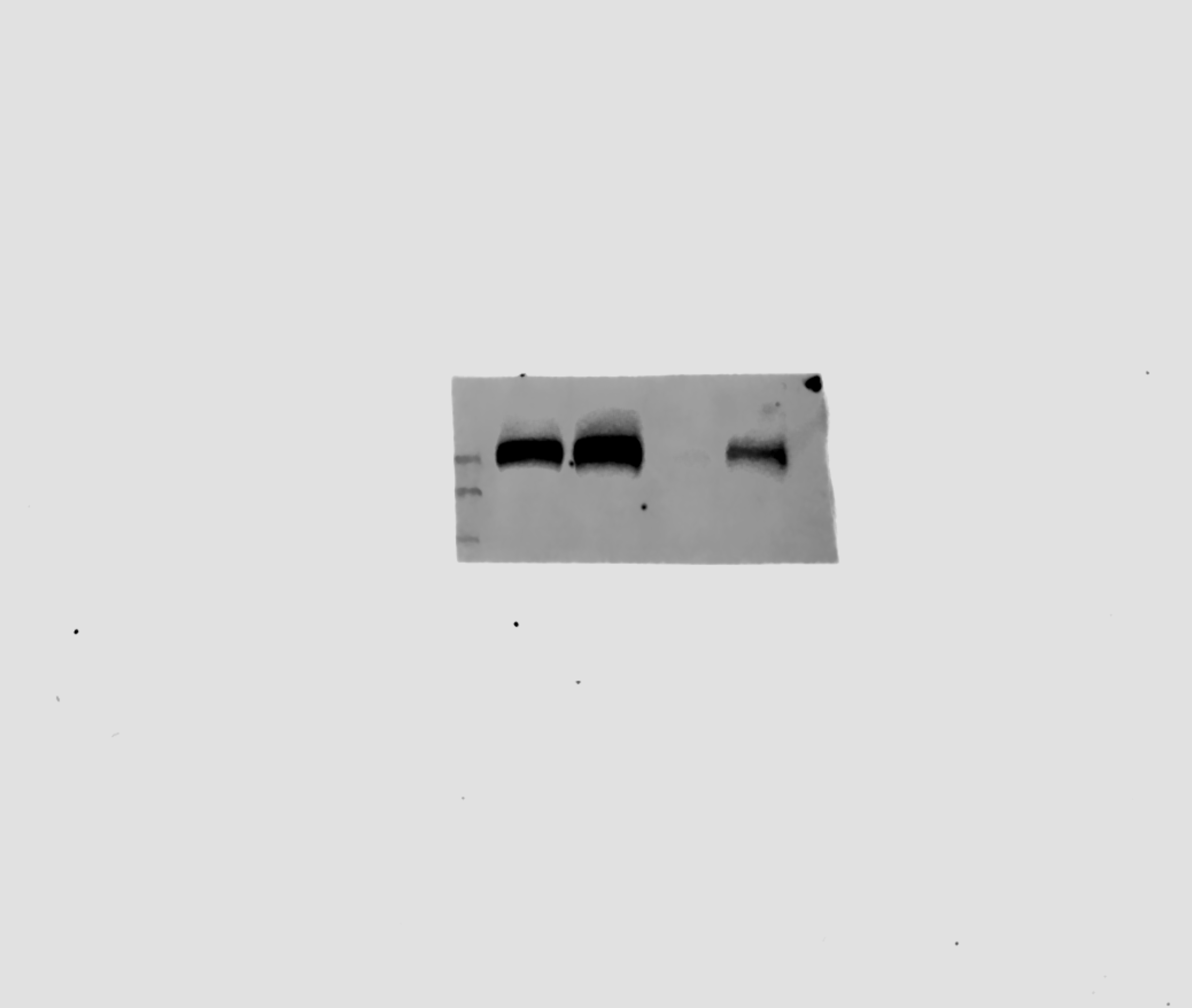

Supplement: Supplementary file 1 [file biomolecules-14-00879-s001.zip › WB_rawdata/Fig6G_nrf2-his.tif]
